# The crystal structure of Ac-AChBP in complex with α-conotoxin LvIA reveals the mechanism of its selectivity towards different nAChR subtypes

**DOI:** 10.1007/s13238-017-0426-2

**Published:** 2017-06-05

**Authors:** Manyu Xu, Xiaopeng Zhu, Jinfang Yu, Jinpeng Yu, Sulan Luo, Xinquan Wang

**Affiliations:** 1 The Ministry of Education Key Laboratory of Protein Science, School of Life Sciences, Beijing Advanced Innovation Center for Structural Biology, Collaborative Innovation Center for Biotherapy, Tsinghua University, Beijing, 100084 China; 2 Key Laboratory of Tropical Biological Resources, Ministry of Education, Key Lab for Marine Drugs of Haikou, Hainan University, Haikou, 570228 China

**Keywords:** base editor, high-fidelity, mouse embryos, proximal-site deamination, whole-genome sequencing

## Abstract

The α3* nAChRs, which are considered to be promising drug targets for problems such as pain, addiction, cardiovascular function, cognitive disorders etc., are found throughout the central and peripheral nervous system. The α-conotoxin (α-CTx) LvIA has been identified as the most selective inhibitor of α3β2 nAChRs known to date, and it can distinguish the α3β2 nAChR subtype from the α6/α3β2β3 and α3β4 nAChR subtypes. However, the mechanism of its selectivity towards α3β2, α6/α3β2β3, and α3β4 nAChRs remains elusive. Here we report the co-crystal structure of LvIA in complex with *Aplysia californica* acetylcholine binding protein (Ac-AChBP) at a resolution of 3.4 Å. Based on the structure of this complex, together with homology modeling based on other nAChR subtypes and binding affinity assays, we conclude that Asp-11 of LvIA plays an important role in the selectivity of LvIA towards α3β2 and α3/α6β2β3 nAChRs by making a salt bridge with Lys-155 of the rat α3 subunit. Asn-9 lies within a hydrophobic pocket that is formed by Met-36, Thr-59, and Phe-119 of the rat β2 subunit in the α3β2 nAChR model, revealing the reason for its more potent selectivity towards the α3β2 nAChR subtype. These results provide molecular insights that can be used to design ligands that selectively target α3β2 nAChRs, with significant implications for the design of new therapeutic α-CTxs.

**Electronic supplementary material:**

The online version of this article (doi:10.1007/s13238-017-0426-2) contains supplementary material, which is available to authorized users.

## Introduction

Neuronal nicotinic acetylcholine receptors (nAChRs) are a group of ligand-gated cation-selective ion channels that play key roles in fast signal transmission in the nervous system (Zoli et al., 2015; Hurst et al., 2013). They are activated by neurotransmitters such as acetylcholine and choline, and they also respond to numerous non-endogenous neuroactive molecules such as nicotine (Cecchini and Changeux, 2015). The nAChRs are implicated in various neurological diseases, including pain, Alzheimer’s and Parkinson’s disease, substance addiction, epilepsy, attention deficit hyperactivity disorder, and depression (Dineley et al., 2015; Le Novere et al., 2002; Laviolette and van der Kooy, 2004), which makes them important drug development targets.

The nAChRs belong to the Cys-loop superfamily of pentameric ligand-gated ion channels (pLGIC), a superfamily which also includes serotonin (5-HT3), gamma-aminobutyric-acid (GABAA and GABAC), and glycine receptors (Ortells and Lunt, 1995). In vertebrates, neuronal nAChRs are composed of α subunits (α2–α10) and β subunits (β2–β4), which combine to form a large array of homo- or hetero-pentamers, such as (α3)_3_(β2)_2_, (α3)_3_(β4)_2_, (α7)_5_, et cetera (Le Novere et al., 2002; Karlin, 2002). Both α and β subunits consist of an extracellular N-terminal ligand-binding domain, four C-terminal transmembrane regions (M1–M4), and an intracellular region extending between M3 and M4 (Hendrickson et al., 2013). Due to sequence and structural homology, especially the high similarity in the ligand-binding site among different nAChRs, the development of novel drugs specific for one type of nAChR or addressing the specificity and selectivity issues of natural agonists and antagonists is a very challenging task, which requires the provision of detailed structural information.

Structural studies of nAChRs have experienced a number of important breakthroughs in the past decades (Karlin, 2002; Unwin, 1995; Brejc et al., 2001; Celie et al., 2004; Morales-Perez et al., 2016). The early results came from low-resolution cryo-EM studies of the *Torpedo* sp. acetylcholine (ACh) receptor (Unwin, 1993, 1995, 2005; Beroukhim and Unwin, 1995; Miyazawa et al., 1999). On the other hand, the crystal structure of the acetylcholine-binding proteins (AChBPs) from mollusks, which are soluble homologs of the extracellular domain (ECD) of nAChR (Brejc et al., 2001; Smit et al., 2001), represented a great leap in the understanding of the structure and function of nAChRs. After this breakthrough, the X-ray crystallographic structures of mouse muscle-type α1 (Dellisanti et al., 2007), two α7 nAChR ECD-AChBPs chimeras (Li et al., 2011; Nemecz and Taylor, 2011), human neuronal α9 nAChR ECD (Zouridakis et al., 2014), and the full-length heteromeric human (α4)_3_(β2)_2_ receptor were determined in quick succession (Morales-Perez et al., 2016). In addition, crystal structures of AChBPs or nAChR ECD in complex with different types of ligands, including AChBPs/nicotine (Celie et al., 2004), AChBPs/α-cobratoxin (Bourne et al., 2005), AChBPs/α-conotoxins (PnIA (A10L, D14K) (Celie et al., 2005), TxIA (A10L) (Dutertre et al., 2007), ImI (Hansen et al., 2005; Ulens et al., 2006), and GIC (Lin et al., 2016)), human α9 nAChR ECD/methyllycaconitine (Zouridakis et al., 2014), and human α2 nAChR ECD/epibatidine (Kouvatsos et al., 2016), provided valuable information for our understanding of receptor-ligand binding specificity and selectivity. Interestingly, despite the only 20%–24% sequence identity with nAChRs, AChBPs display a striking structural resemblance to the nAChR ECD, and their pharmacological properties closely resemble those of nAChRs. Therefore, AChBPs still represent the best template for the characterization of ligand binding to the extracellular ligand-binding domain of nAChRs (Cecchini and Changeux, 2015).

Conotoxins are disulfide-bridged peptides isolated from cone-snail venom, which act on a wide range of ion channels, including voltage-gated sodium, potassium and calcium channels as well as nAChRs (Lebbe et al., 2014). To date, five types of conotoxins, termed α, δ, κ, μ, and ω, have been isolated and characterized, and each type attacks a different target, whereby α-conotoxins mainly inhibit nAChRs (Tsetlin et al., 2009). The α-conotoxins display a consensus fold with a central helical region braced by two conserved disulfide bridges (Azam and McIntosh, 2009). Based on the number of residues between the second and third, as well as between the third and fourth cysteine residues, α-conotoxins are classified into different families, such as α3/5, α4/3, α4/6, α4/7 et cetera (Mir et al., 2016). As one of the largest and most diverse groups of nAChR antagonists, they have tremendous therapeutic potential for the treatment of various neurological diseases, including epilepsy and neuropathic pain (Tsetlin et al., 2009; Azam and McIntosh, 2009).

Owing to their relatively rigid framework structure, combined with great diversity at the amino-acid sequence level, α-conotoxins bind to distinct nAChR subtypes with different selectivity (Rucktooa et al., 2009), which makes them remarkable probes for structural studies. Because ligands that selectively inhibit α3β2, α6/α3β2β3, and α3β4 nAChRs are lacking, in an earlier study we investigated the α4/7 conotoxin LvIA, which is the first α-conotoxin discovered from the carnivorous marine gastropod *Conus lividus* (Luo et al., 2014). It has high affinity for α3β2 nAChRs with an IC_50_ of 8.7 nmol/L, and it is notable for being their most selective known probe, as it can distinguish the α3β2 nAChRs from the α6/α3β2β3 (IC_50_ 108 nmol/L) and α3β4 nAChR (IC_50_ 148 nmol/L) subtypes (Luo et al., 2014). As α3* nAChRs are likely to modulate pain sensation and cardiovascular function, and the α3 subunit is structurally very closely related to α6 (Salas et al., 2009; Paolini and De Biasi, 2011), strategies to selectively distinguish between the α3* and α6* subunits, as well as to modulate the function of α3* nAChRs are of great importance.

To reveal the mechanism responsible for the distinctive binding profile and selectivity of LvIA towards different α3*nAChRs, we solved the crystal structure of α-conotoxin LvIA in complex with the acetylcholine binding protein from *Aplysia californica* (Ac-AChBP) at 3.4 Å resolution. Based on this complex structure, together with homology models based on other nAChR subtypes, as well as binding affinity assays, we offer an explanation for its binding features, which has significant implications for the design of new therapeutic α-conotoxin derivatives.

## Results

### Overall structure of the Ac-AChBP complex

**Figure 1:**
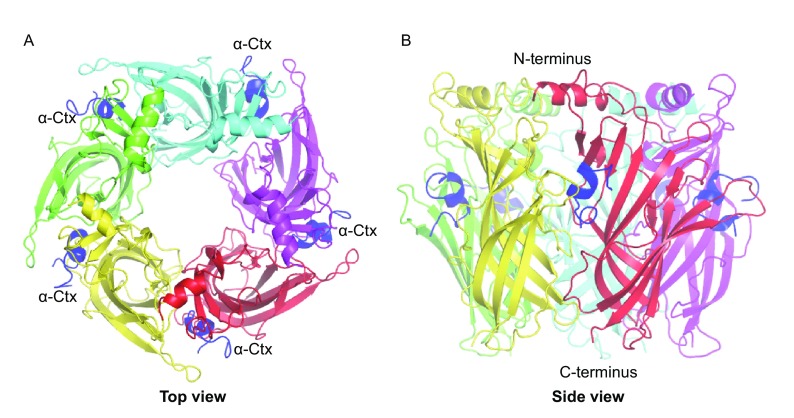
The X-ray crystal structure of α-conotoxin LvIA binding to Ac-AChBP. (A) The top view of Ac-AChBP/LvIA structure, showing LvIA (blue) in five binding sites. (B) The side view of Ac-AChBP in complex with LvIA, two adjacent protomers of the pentamer with a bound α-conotoxin LvIA molecule (in blue)

We solved the crystal structure of Ac-AChBP in complex with α-CTx LvIA at 3.4 Å resolution, using the molecular replacement method (Table 1). The complex displays a striking structural resemblance to earlier Ac-AChBP/α-CTx structures that were solved before. Upon structural superimposition, the Ac-AChBP/LvIA complex structure had a RMSD of 0.72 Å for all paired Cα atoms compared with the Ac-AChBP/PnIA (A10L, D14K) complex (PDB code 2BR8) (Celie et al., 2005), 0.52 Å with the Ac-AChBP/ImI complex (PDB code 2C9T and 2BYP) (Hansen et al., 2005; Ulens et al., 2006), 0.92 Å with the Ac-AChBP/TxIA (A10L) complex (PDB code 2UZ6) (Dutertre et al., 2007), 0.78 Å with the Ac-AChBP/BuIA complex (PDB code 4EZ1) and 0.34 Å with the Ac-AChBP/GIC complex (PDB code 5CO5) (Lin et al., 2016). The protein forms a windmill-like pentamer along a five-fold axis, forming five highly similar ligand-binding sites between two adjacent protomers (Fig. 1). Upon binding, α-CTxs are buried in the five ligand-binding sites (Fig. 1A). α-CTx LvIA is a C-terminally-amidated peptide comprising 16 amino acids with two disulfide bridges. In the ligand-binding site, it shares a common orientation with other previously determined α-CTxs (Fig. S1), with its central helix protruding into the binding site and the N- and C-termini located at the bottom and top of the binding site, respectively (Fig. 1B)

**Table 1:** Crystal diffraction data collection and structural refinement statistics

Data collection	
Beamline	SSRF BL17U
Wavelength	0.9796 Å
Space group	*P*2_1_2_1_2_1_
Cell dimensions	
a, b, c (Å)	77.39, 83.99, 209.68
α, β, γ (°)	90, 90, 90
Resolution (Å)	27.71–3.44 (3.56–3.44)
*R*_merge_ (%)	17.0 (87.6)
*I*/δ*I*	8.5 (3.0)
Completeness (%)	98
Redundancy	5.0 (5.1)
Refinement	
Resolution (Å)	27.71–3.44 (3.56–3.44)
No. reflections	18405 (1463)
*R*_work_/*R*_free_ (%)	23.9/28.3
No. atoms	8833
B-factors (Å^2^)	94.75
r.m.s. deviations	
Bond lengths (Å)	0.004
Bond angles (°)	0.66
Ramachandran plot (%)	
Favored	98.45
Allowed	1.55
Disallowed	0

### Structural basis for interactions between the α-CTxs LvIA and Ac-AChBP

**Figure 2:**
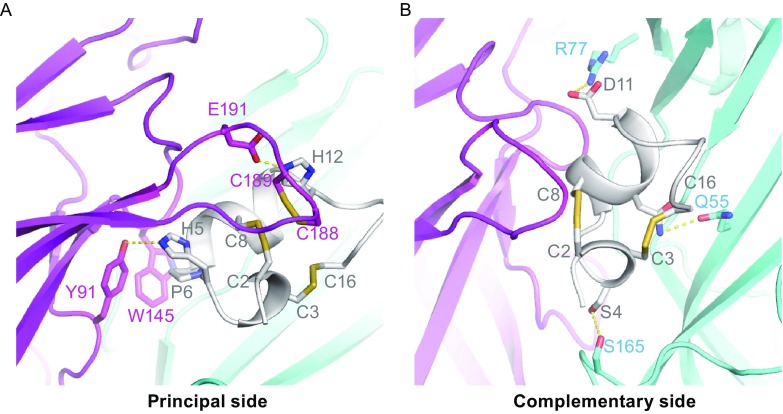
Binding interface between α-conotoxin LvIA and Ac-AChBP. (A) The disulfide bridge packing and hydrogen-bonding interactions (represented by yellow dashed line) on the principal side. Residues His-5 and His-12 of the LvIA form hydrogen bonds with Tyr-91 and Glu-191 of the Ac-AChBP, respectively. Disulfide bond C2-C8 in the LvIA closely packed together with C188-C189 in the Ac-AChBP. (B) On the complementary binding side, Ser-4 and Asn-9 of LvIA forms a hydrogen bond (represented by yellow dashed line) with Ser-165 and Gln-55 of Ac-AChBP, respectively. Electronic interaction was also observed between Asp-11 of LvIA and Arg-77 of Ac-AChBP

Each α-CTx molecule interacts with two adjacent Ac-AChBP protomers at their interface, forming the principal and complementary binding sides. Most interactions on the principal side were between the peptide and the C-loop (Gln-184~Tyr-193) of one of the Ac-AChBP protomers. Upon binding to LvIA, the C-loop has a significant conformational change that was also observed in the complex structures of Ac-AChBP with other α-CTxs (Fig. S2). Residues His-5, Pro-6, Ala-7, and His-12 of α-CTx LvIA play key roles on the principal binding site. His-5 forms a hydrogen bond with Tyr-91, Pro-6 undergoes a hydrophobic interaction with Tyr-91 and Trp-145, Ala-7 displays extensive hydrophobic interactions with Trp-145, Val-146, Tyr-147, and Tyr-193, while a salt bridge between His-12 of α-CTx LvIA and Glu-191 of Ac-AChBP was also observed. In addition, the Cys-2/Cys-8 disulfide bridge of the peptide was found to be stacked against the vicinal Cys-188/Cys-189 disulfide bond of Ac-AChBP (Fig. 2A).

The complementary binding side is situated at the interface between the LvIA and the β-sheet of another Ac-AChBP protomer. On the complementary binding side, Ser-4 and Asn-9 of LvIA form hydrogen bonds with Ser-165 and Gln-55 of Ac-AChBP, respectively. An electronic interaction was also observed between Asp-11 of LvIA and Arg-77 of Ac-AChBP. Overall, the complementary side does not seem to play a significant role in the interaction between the peptide and the Ac-AChBP (Fig. 2B).

### Binding assay of LvIA with Ac-AChBP

To validate our co-crystal structure and identify residues that confer potency for Ac-AChBP, we conducted surface plasmon resonance (SPR) analysis to study the binding affinities of LvIA and its mutants. The results are summarized in Table 2. The wild-type LvIA and its analogues were produced using solid-phase peptide synthesis. Among the studied mutants, H5A, P6A, and H12A completely lost their binding capacity for Ac-AChBP. The other substitutions, such as Ac-AChBP S4A, exhibited comparatively small decreases of binding affinity. The most notable changes were the improved binding affinities of LvIA (N9A) and LvIA (D11A). LvIA (N9A) bound Ac-AChBP with a *K*_d_ value of 82.78 nmol/L, which represents a 1.6-fold higher affinity than native LvIA. LvIA (D11A) had a *K*_d_ value of 17.48 nmol/L for Ac-AChBP, which means that its potency had increased 7.5-fold.

**Table 2:** Binding affinities of α-conotoxin LvIA and its mutants towards Ac-AChBP

Peptide	Sequence	*K*_d_ (nmol/L)	Ratio
LvIA WT	GCCSHPACNVDHPEIC*	131.6	1
LvIA (S4A)	GCCAHPACNVDHPEIC*	23.83	0.18
LvIA (H5A)	GCCSAPACNVDHPEIC*	ND	ND
LvIA (P6A)	GCCSHAACNVDHPEIC*	ND	ND
LvIA (A7G)	GCCSHPGCNVDHPEIC*	ND	ND
LvIA (N9A)	GCCSHPACAVDHPEIC*	82.78	0.63
LvIA (D11A)	GCCSHPACNVAHPEIC*	17.48	0.13
LvIA (H12A)	GCCSHPACNVDAPEIC*	ND	ND

*ND* not determined

Asterisks indicate an amidated C terminusc

### Homology modeling of rat α3β2, α6β2, and α3β4 nAChRs and docking with LvIA

**Figure 3:**
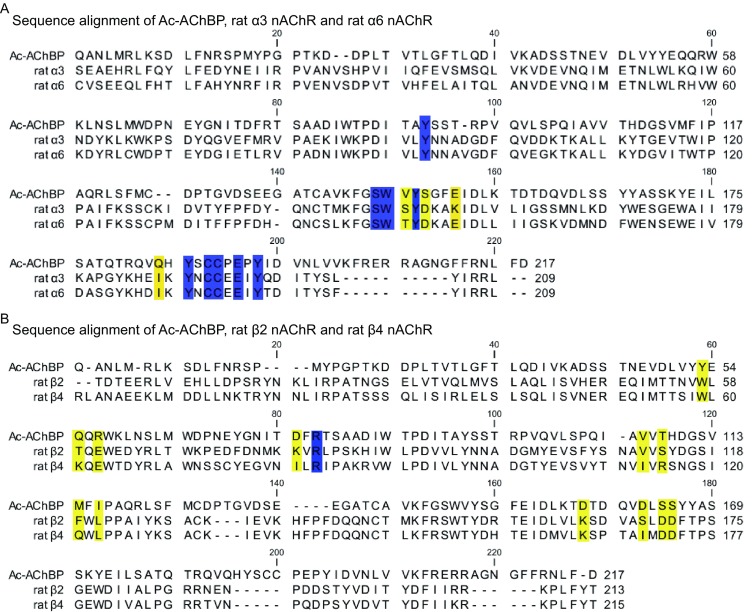
Primary sequence comparison of Ac-AChBP, and the rat α3, α6, β2, and β4 nAChRs. (A) Sequence alignment of Ac-AChBP, rat α3 nAChR, and rat α6 nAChR; regions colored yellow indicate the amino acids that are not conserved between the α3 and α6 ligand binding sites, regions colored blue indicate the amino acids that are conserved between the Ac-AChBP, α3 nAChR, and α6 nAChR ligand binding sites. (B) Sequence alignment of Ac-AChBP, rat β2 nAChRs, and rat β4 nAChRs; regions colored with yellow indicate the amino acids that are not conserved between the α3 and α6 ligand binding sites, regions colored blue indicate the amino acids that are conserved between the Ac-AChBP, β2 nAChR, and β4 nAChR ligand binding sites

LvIA exhibits a high affinity towards the rat α3β2 nAChR (8.67 nmol/L), but has a 13-fold lower affinity towards the rat α6/α3β2β3 nAChRs (108 nmol/L) and a 17-fold lower affinity towards the rat α3β4 subtype (148 nmol/L). To gain molecular insights into the interactions of LvIA with the α3β2, α6β2, and α3β4 nAChR subtypes, homology models of the extracellular ligand binding domain of rat α3β2, α6β2, and α3β4 nAChRs bound to LvIA were constructed using the co-crystal structure of Ac-AChBP/LvIA as template. Homology modeling has been used in many publications examining conotoxins and nAChRs (McDougal et al., 2013; Luo et al., 2010; Sambasivarao et al., 2014). The interacting residues between LvIA and Ac-AChBP, rat α3β2, rat α6β2, and rat α3β4 nAChRs are listed in Table 3. A sequence alignment of Ac-AChBP with the rat α3, α6, β2, and β4 nAChRs is shown in Fig. 3. Asterisks (*) indicate amino acids that are not conserved between the α3 and α6, or β2 and β4 ligand-binding sites.

**Table 3:** Contacts between residues of LvIA and those of Ac-AChBP, α3β2 nAChR, α6β2 nAChR and α3β4 nAChR, respectively

LvIA/Ac-AChBP crystal structure		LvIA/α3β2 nAChR model		LvIA/α3β4 nAChR model		LvIA/α6β2 nAChR model	
Ac-AChBP	LvIA	α3β2nAChRs	LvIA	α3β4nAChRs	LvIA	α6β2nAChRs	LvIA
Principal side							
Tyr-91	His-5, Pro-6	Tyr-93	His-5, Pro-6	Tyr-93	His-5, Pro-6	Tyr-93	His-5, Pro-6
Ser-144		Ser-148	Ala-7	Ser-148	Ala-7		
Trp-145	Pro-6, Ala-7	Trp-149	Pro-6, Ala-7	Trp-149	Pro-6, Ala-7	Trp-149	Pro-6, Ala-7
Val-146	Ala-7	Ser-150	Asp-11	Ser-150	Asp-11	Thr-150	Ala-7
Tyr-147	Ala-7	Tyr-151	Ala-7	Tyr-151	Ala-7	Tyr-151	Ala-7
Ser-148	Asp-11						
Glu-151	Asp-11	Lys-155	Asp-11	Lys-155	Asp-11	Glu-155	Asp-11
Gln-184	His-5						
Tyr-186	Gly-1, Cys-2, His-5	Tyr-190	Gly-1, Cys-2, His-5	Tyr-190	Gly-1, Cys-2, His-5	Tyr-190	Cys-2, His-5
Cys-188,	Cys-2, Ile-15					Cys-192	Cys-2, Ile-15
Cys-189	Cys-2, Cys-8, His-12	Cys-193	Cys-8, His-12	Cys-193	Cys-8, His-12	Cys-193	His-12
Glu-191	His-12	Glu-195	His-12	Glu-195	His-12	Glu-195	His-12
Tyr-193	His-5, Ala-7, Cys-8, Asp-11, His-12	Tyr-197	His-5, Ala-7, Cys-8, Asp-11, His-12	Tyr-197	His-5, Ala-7, Cys-8, Asp-11, His-12	Tyr-197	His-5, Ala-7, Cys-8, Asp-11
Complementary side							
		Met-36	Asn-9	Glu-38	Cys-16	Met-36	Asn-9
Thr-34		Ser-38	Ser-4			Ser-38	Ser-4
Tyr-53	Ser-4	Trp-57	Ser-4, Pro-6	Trp-59	Pro-6	Trp-57	Ser-4, Pro-6
Gln-55	Asn-9, Cys-16	Thr-59	Asn-9	Lys-61	Asn-9, Cys-16	Thr-59	Asn-9
Arg-57	Cys-16	Glu-61	Cys-16	Glu-63	Pro-13	Glu-61	Cys-16
Asp-75		Lys-79	Asp-11			Lys-79	Asp-11
Arg-77	Asp-11	Arg-81	Asp-11	Arg-83	Asp-11	Arg-81	Asp-11
Val-106	Val-10	Val-111	Val-10	Ile-113	Val-10	Val-111	Val-10
Thr-108				Arg-115	Pro-13		
Met-114	Asn-9, Val-10, Pro-13	Phe-119	Asn-9, Val-10	Gln-121	Val-10, Pro-13	Phe-119	Asn-9, Val-10
Ile-116	Pro-6, Asn-9, Val-10			Leu-123	Val-10		
Asp-157	Cys-16	Lys-163	Pro-13, Cys-16	Lys-165	Pro-13, Cys-16	Lys-163	Pro-13, Cys-16
Asp-162	Cys-3, Ser-4	Ser-168	Ser-4	Ile-170	Cys-3, Ser-4	Ser-168	Ser-4
Ser-164	Gly-1, Ser-4	Asp-170	Gly-1	Asp-172	Gly-1, Ser-4	Asp-170	Gly-1
Ser-165	Ser-4	Asp-171	Ser-4	Asp-173	Ser-4	Asp-171	Ser-4

**Figure 4:**
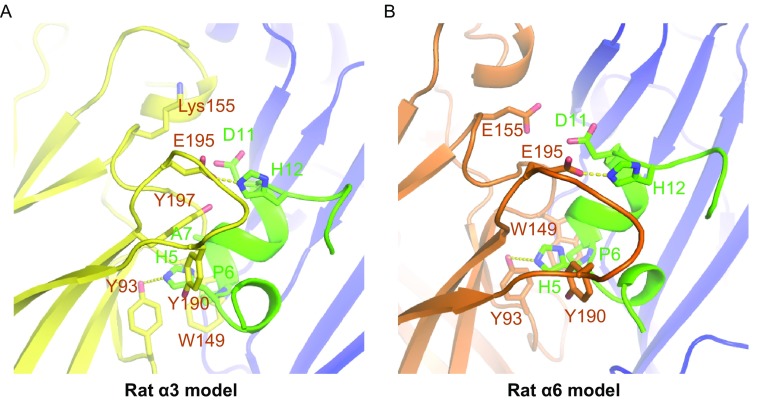
Homology modelling of rat α3 and rat α6 bound with α-conotoxin LvIA using Ac-AChBP/LvIA as the template. (A) In the rat α3 homology model, the Pro-6 of α-CTx LvIA exhibits very strong hydrophobic interactions with the Trp-149 of α3 and Trp-57 of β2 subunit both on the principal and complementary binding side; His-5 of LvIA has a hydrogen bond with Tyr-193 and widely hydrophobic interactions with Tyr-93, Tyr-190, and Tyr-197 of α3 subunit; Ala-7 exhibits hydrophobic interactions with Trp-149, Tyr-151, and Tyr-197 of α3 subunit and His-12 of LvIA forms a salt bridge with Glu-195 of α3 subunit. (B) In the rat α6 homology model, contacts were very similar with the α3 model except Lys-155 in α3 subunit is replaced by Glu-155 and cause an electrostatic repulsion with Asp-11 of LvIA

**Figure 5:**
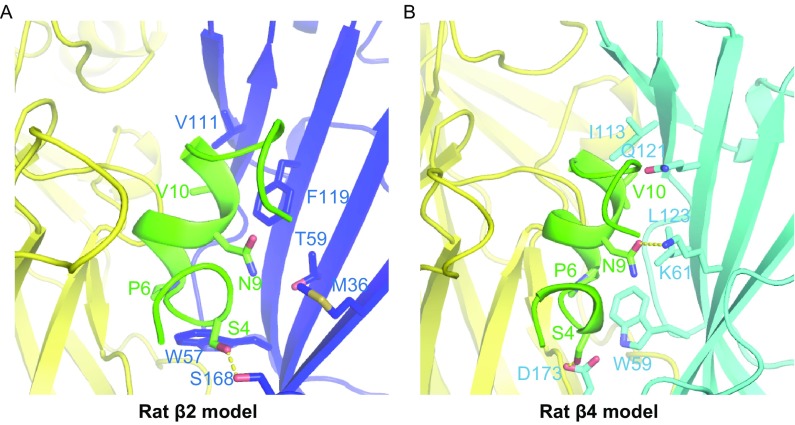
Homology modelling of rat β2 and rat β4 bound with α-conotoxin LvIA using Ac-AChBP/LvIA as the template. (A) In the rat β2 homology model, Asn-9 resides in a hydrophobic pocket that is formed by Met-36, Thr-59, and Phe-119; Val-10 performs a hydrophobic interaction with Val-111. (B) In rat β4 homology model, there is a little positional shift of Asn-9, making it form a hydrogen bond with Lys-61 but losing extensively hydrophobic interactions with Ile-123, Gln-121, and Leu-123

In the homology model of LvIA bound to the rat α3β2 nAChR, we noticed that Pro-6 of α-CTx LvIA exhibits very strong hydrophobic interactions with Trp-149 of α3 and Trp-57 of the β2 subunit, on the principal and complementary binding sides, respectively. His-5 of LvIA has a hydrogen bond with Tyr-193 and extensive hydrophobic interactions with Tyr-93, Tyr-190, and Tyr-197 of the α3 subunit. Ala-7 exhibits hydrophobic interactions with Trp-149, Tyr-151, and Tyr-197 of the α3 subunit, whereas His-12 of LvIA forms a salt bridge with Glu-195 of the α3 subunit (Fig. 4A and Table 2). These interactions were very similar to the contacts observed in the Ac-AChBP/LvIA crystal structure (Table 2). However, Asn-9 lies within a hydrophobic pocket that is formed by Met-36, Thr-59, and Phe-119 of β2 subunit (Fig. 5A), which was different from the hydrogen-bond contact between Ac-AChBP and LvIA.

A comparison of the α3β2 and α6β2 nAChR models allowed us to pinpoint the key amino acid residues on the principal side which is responsible for increased binding of LvIA to the α3β2 vs. the α6β2 subtype (Fig. 4). One notable change is the substitution of Lys-155 in the α3 subunit with Glu-155 in the α6 subunit. Lys-155 in the α3 subunit, which is a positively charged residue, forms a salt bridge with the negatively charged Asp-11 of LvIA. However, in the α6 subunit, Lys-155 is replaced by Glu-155, which causes an electrostatic repulsion of the Asp-11 from LvIA. This phenomenon alone may account for a large part of the observed selective potency towards the α3β2 as opposed to the α6β2 nAChR subtype.

Additionally, a comparison of the α3β2 and α3β4 nAChR models allowed us to determine the key residues on the complementary side responsible for increased binding of LvIA to the α3β2 vs. the *α*3β4 subtype (Fig. 5). We previously reported mutational studies conducted to assess the influence of residues from the β2 subunit versus those from the β4 subunit on the binding of α-CTx LvIA (Zhangsun et al., 2015). Two β2 mutations, α3β2 (F119Q) and α3β2 (T59K), strongly enhanced the binding affinity of LvIA (IC_50_ 0.58 nmol/L and IC_50_ 0.96 nmol/L, respectively), and one β2 mutation, α3β2 (V111I), substantially reduced the affinity (IC_50_ 126 nmol/L) (Zhangsun et al., 2015). According to our α3β2 model, Asn-9 lies within a hydrophobic pocket that is formed by Met-36, Thr-59, and Phe-119, whereas Val-10 undergoes a hydrophobic interaction with Val-111. The side chains of the α3β2 (T59K) and α3β2 (F119Q) nAChRs increase the polar contact between LvIA and the β2 subunit, forming two hydrogen bonds with Asn-9 of LvIA, which explains the improved binding affinity of LvIA towards the α3β2 (T59K) and α3β2 (F119Q) nAChR subtypes. Substitution of valine with isoleucine in the α3β2 (V111I) nAChR subtype may cause a steric clash between Val-10 and β2 V-111, explaining the decreased binding affinity of LvIA. However, in our α3β4 nAChR model, there is only a small positional shift of Asn-9, making it form a hydrogen bond with Lys-61, but also lose the extensive hydrophobic interactions with Ile-123, Gln-121, and Leu-123. This may explain why LvIA is more potent towards α3β2, demonstrating that hydrophobic interactions are crucial for the potency of LvIA when acting on the α3β2 and α3β4 nAChR subtypes.

## Discussion

Analysis of the precise role of α3* nAChRs has been hampered by the lack of specific molecular probes. The α-conotoxin LvIA was discovered and characterized in 2014 (Luo et al., 2014). It has a high affinity for the α3β2 nAChR with an IC_50_ of 8.7 nmol/L, and is selective for the α3β2 nAChR subtype over the α3/α6β2β3 and α3β4 subtypes. The previously characterized conotoxins that block α3β2 nAChRs offer only poor selectivity towards α3β2 vs. α6β2* nAChRs (Luo et al., 2014). However, the expression patterns of α3β2* nAChR and α6β2* nAChRs overlap in dopaminergic regions, where α6β2* nAChRs predominate. LvIA may therefore be a highly valuable probe to interrogate the function and significance of α3β2 nAChRs in normal and disease physiology. In this study, we solved a co-crystal structure of Ac-AChBP/LvIA, and together with LvIA docking on different nAChR subtypes, concluded that Asn-9 and Asp-11 of α-CTx LvIA are the key residues responsible for its selectivity. According to molecular docking results, the Asp-11 residue of LvIA can make a salt bridge with Lys-155 of the rat α3 subunit, whereas it is electrostatically repulsed by Glu-155 of the rat α6 subunit, explaining the large difference in affinity towards the α3β2 and α6β2 nAChR subtypes. Asn-9 lies within a hydrophobic pocket that is formed by Met-36, Thr-59, and Phe-119 in the α3β2 nAChR model, revealing the reason for its more potent selectivity towards the α3β2 nAChR subtype.

Our results also confirm the results reported by Hone et al. that an N11R substitution in α-CTx PeIA essentially abolished the activity of PeIA for α3β2 but not for α6/α3β2β3 nAChR subtypes (Hone et al., 2013). PeIA (N11R) has a positively charged amino acid in the 11th position, while LvIA has a negatively charged residue in its place. Their homology models indicate that PeIA(N11R)-binding is disfavored in α3-containing nAChRs, potentially due to a repulsive charge-charge interaction with Lys152 from the rat α3 subunit (in this publication, Lys152 is equal to Lys155 due to a different numbering system), which was in good agreement with our experimental results. In conclusion, charged residues in the 11th position of the 4/7 α-CTx might be important determinants of binding to α6 and α3 subunits.

Taken together, our findings increase the understanding of the interactions between the α-CTx LvIA and various nAChR subtypes. We identified key residues, such as His-5, Pro-6, Asn-9, Asp-11, and His-12, that are involved in toxin-receptor interaction. This information will be valuable in the design and development of potent α3β2-selective drugs, with significant implications for the treatment of neuropathic pain and nicotine addiction.

## Materials and methods

### Peptide synthesis

We used regio-selective disulfide bond formation with Acm-protected cysteine residues incorporated at positions 1 and 3, and a two-step oxidation procedure, to produce the alanine mutant peptides in a globular conformation (I–III and II–IV disulfide bonds). Briefly, the first disulfide bridge was closed using 20 mmol/L potassium ferricyanide in 0.1 mol/L Tris-HCl, pH 7.5. The solution was allowed to react for 45 min, and the monocyclic peptide was purified by reverse-phase HPLC. Simultaneous removal of the acetamidomethyl groups and closure of the second disulfide bridge was accomplished via oxidation, by combining the monocyclic peptide in the HPLC eluent with an equal volume of 10 mmol/L iodine in H_2_O/trifluoroacetic acid/acetonitrile (78:3:25 by volume) and allowing it to react for 10 min. The reaction was terminated by the addition of ascorbic acid, diluted 20-fold with 0.1% (*v*/*v*) trifluoroacetic acid, and the bicyclic product purified by reverse-phase HPLC, same as above. The masses of the peptides were verified by matrix-assisted laser desorption ionization time-of-flight (MALDI-TOF) mass spectrometry.

### Protein expression and purification

Ac-AChBP was overexpressed in High Five insect cells maintained in SIM-HF medium (Sino Biological Inc., China) using the Bac-to-Bac baculovirus expression system (Invitrogen, Thermo Fisher Scientific, USA). Sf9 insect cells were maintained in Insect-XPRESS™ Protein-free Insect Cell Medium (Lonza, Switzerland). The cDNA encoding the full-length Ac-AChBP was cloned into the pFastBac-Dual vector (Invitrogen) with a C-terminal 6× His tag to facilitate purification. The plasmid was used to transform competent *E*. *coli* DH10 Bac cells, and the extracted bacmid was used to transfect Sf9 cells using the Cellfectin II Reagent (Invitrogen). The low-titer virions (P0) were harvested after incubation of the transfected cells at 26°C for 7–9 days and amplified to generate high-titer virus stock. An aliquot comprising 10 mL of the amplified high-titer virus (P1) was used to infect cultures comprising 1 L of High Five insect cells at a density of 2 × 10^6^ cells/mL. The culture supernatants containing soluble Ac-AChBP were harvested by centrifugation at 4,000 rpm for 15 min at 4°C, 48–72 h after infection. The culture supernatants were further concentrated and buffer-exchanged to HBS (10 mmol/L Hepes, pH 7.2, 150 mmol/L NaCl) using a 30 KD ultrafiltration cartridge. Ac-AChBP was captured on Nickel-NTA resin (GE Healthcare, USA) and eluted with 500 mmol/L imidazole in HBS buffer. Further purification was performed by gel-filtration chromatography on the Superdex 200 10/300 High Performance column (GE Healthcare, USA) with protein solution as mobile phase at 0.5 mL/min.

### Crystallization and data collection

Purified Ac-AChBP and synthesized α-CTx LvIA were mixed at a molar ratio of 1:1.5 at 4°C. After incubation for 2 h, the sample was loaded onto a Superdex 200 10/300 High Performance column (GE Healthcare). The peak fractions were collected and concentrated to ~20 mg/mL in HBS buffer for crystallization. Crystals were successfully grown at 18°C using the sitting drop vapor diffusion method by mixing equal volumes of protein and reservoir solution. Crystals of Ac-AChBP/LvIA grew in buffer containing 1.2 mol/L DL-Malic acid pH 7.0, 0.1 mol/L BIS-TRIS propane pH 7.0. Prior to data collection, the crystals were cryocooled in liquid nitrogen, using reservoir solution plus 20% (v/v) glycerol as cryoprotectant. Diffraction data were collected at the BL17U beam line of the Shanghai Synchrotron Research Facility (Shanghai, China). Diffraction data were indexed, integrated and scaled using HKL2000 (Otwinowski and Minor, 1997).

### Structure determination and refinement

The structure was solved via molecular replacement using the PHASER crystallographic software with Ac-AChBP/GIC (PDB code 5CO5) as search model (McCoy et al., 2007). The model was further rebuilt in COOT (Emsley and Cowtan, 2004) and refined in PHENIX (Adams et al., 2002). Structure validation was performed with the program PROCHECK (Laskowski et al., 1993), and all structural figures were generated using PYMOL (http://www.pymol.org/). Data collection and structure refinement statistics are summarized in Table 1.

### SPR analysis

Real-time binding analysis using surface plasmon resonance (SPR) was conducted on a Biacore S200 instrument (GE Healthcare, USA) at 25°C. Ac-AChBP was immobilized on a research-grade CM5 sensor chip (GE Healthcare, USA) using the amine-coupling method. Flow cell 1 was left blank as a reference. Ac-AChBP (20 µg/mL) in 10 mmol/L sodium acetate pH 5.0 was immobilized to 600 response units in flow cell 2. For the collection of data, α-CTx LvIA and its mutants were injected into the flow cells in a buffer comprising 10 mmol/L HEPES pH 7.2, 150 mmol/L NaCl, and 0.005% (*v*/*v*) Tween-20 at various concentration using a 30 μL/min flow rate. Data were analyzed using the Biacore S200 evaluation software by fitting to a 1:1 Langmuir binding model.

### Homology modelling and docking

All the modelling and docking were performed in Discovery Studio Client 4.0 (Accelrys, San Diego, CA). The molecular models of extracellular ligand-binding domains of the rat nAChRs such as α3β2, α6β2, and α3β4 were generated based on the template of Ac-AChBP structure using the homology modelling program Modeler (Webb, 2014). The LvIA docking was based on the reference model of the Ac-AChBP/LvIA complex. The models were refined with a side-chain refinement and energy minimization process. All modelling and docking structures were verified by the program Profiles-3D in the Discovery Studio platform, as well as by the MolProbity server (Davis et al., 2007; Davis et al., 2004).

### PDB deposition

The coordinates and diffraction data have been deposited into the Protein Data Bank with accession code 5XGL.

## Electronic supplementary material

Below is the link to the electronic supplementary material.

Supplementary material 1 (PDF 661 kb)
