# Erratum to: The crystal structure of Ac-AChBP in complex with α-conotoxin LvIA reveals the mechanism of its selectivity towards different nAChR subtypes

**DOI:** 10.1007/s13238-017-0478-3

**Published:** 2017-09-23

**Authors:** Manyu Xu, Xiaopeng Zhu, Jinfang Yu, Jinpeng Yu, Sulan Luo, Xinquan Wang

**Affiliations:** 1 The Ministry of Education Key Laboratory of Protein Science, School of Life Sciences, Beijing Advanced Innovation Center for Structural Biology, Collaborative Innovation Center for Biotherapy, Tsinghua University, Beijing, 100084 China; 2 Key Laboratory of Tropical Biological Resources, Ministry of Education, Key Lab for Marine Drugs of Haikou, Hainan University, Haikou, 570228 China

## Erratum to: Protein Cell (2017) 8(9):675–685 DOI 10.1007/s13238-017-0426-2

In the original publication of the article the keywords are incorrectly online published. The correct keywords should read as “α-conotoxin, nicotinc acetylcholine receptor, acetylcholine binding protein, X-ray crystallography”.

