# Supplementary material for: The crystal structure of Ac-AChBP in complex with α-conotoxin LvIA reveals the mechanism of its selectivity towards different nAChR subtypes
Source: Protein Cell. 2017 Jun 5;8(9):675–85. doi: 10.1007/s13238-017-0426-2 (PMC5563285; doi:10.1007/s13238-017-0426-2)

The crystal structure of Ac-AChBP in complex with α-conotoxin LvIA reveals the mechanism of its selectivity towards different nAChR subtypes

Manyu Xu[1#], Xiaopeng Zhu[2#], Jinfang Yu[1], Dongting ZhangSun[2], Yong Wu[2], Sulan Luo[2,*], Xinquan Wang[1,*]

1. The Ministry of Education Key Laboratory of Protein Science, Beijing Advanced Innovation Center for Structural Biology, Collaborative Innovation Center for Biotherapy, School of Life Sciences, Tsinghua University, Beijing, 100084, P.R. China.
2. Key Laboratory of Tropical Biological Resources, Ministry of Education, Hainan University, Haikou, Hainan, 570228, P.R. China.

[#]Equal contribution

*Correspondence to Sulan Luo (luosulan2003@163.com) and Xinquan Wang (xinquanwang@mail.tsinghua.edu.cn)

Running Title: Co-crystal structure of α-conotoxin LvIA and acetylcholine binding proteins

Figure S1: **Supplementary Figure 1: Backbone orientations observed in co-crystal structures of Ac-AChBP with five α-conotoxins. (A)** The backbone of LvIA is shown in light blue, BuIA in red, TxIA (A10L) in green, PnIA (A10L, D14K) in blue, ImI in yellow and GIC in magenta. **(B)** Multiple sequence alignment of α-conotoxins LvIA, BuIA, TxIA (A10L), PnIA (A10L, D14K), ImI and GIC. Disulfide bridges between Cys2-Cys8 and Cys3-Cys16 are shown in red.

A

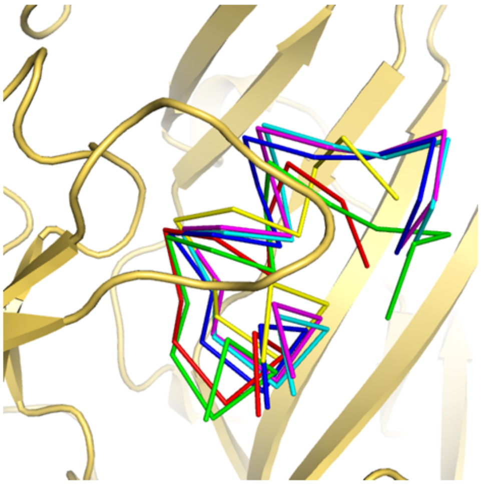

B

disulfide

α-Conotoxin	Sequence
LvIA	G C C S H P A C N V D H P E I C
BuIA	G C C S T P P C A V L Y . . . C
TxIA (A10L)	G C C S R P P C I L N N P D L C
PnIA (A10L, D14K)	G C C S L P P C A L N N P K Y C
ImI	G C C S D P R C A W R C
GIC	G C C S H P A C A G N N Q H I C

Figure S2: Crystal structures of Ac-AChBP in different comformation. (A) Top view of Ac-AChBP/HEPES structure, showing Loop C in close status. **(B)** Top view of Ac-AChBP/LvIA structure, showing Loop C in open status. **(C)** Top view of Ac-AChBP/GIC structure, showing Loop C in close status.

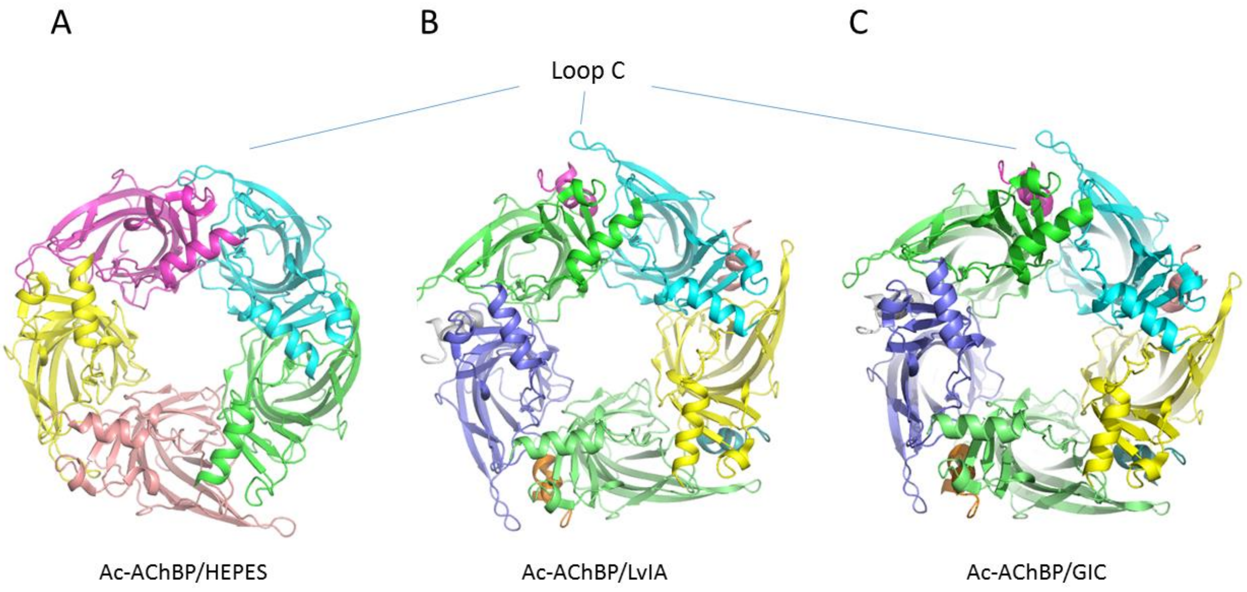

Supplement: Supplementary file 1 — Supplementary material 1 (PDF 661 kb) [file 13238_2017_426_MOESM1_ESM.pdf]
